# Emerging Infections: A Guide to Diseases, Causative Agents, and Surveillance

**DOI:** 10.3201/eid2010.141110

**Published:** 2014-10

**Authors:** Frank J. Sorvillo

**Affiliations:** Associate Editor, Emerging Infectious Diseases;; University of California at Los Angeles School of Public Health, Los Angeles, California, USA

**Keywords:** emerging infections, emerging infectious diseases, causative agents, surveillance

Given the importance of emerging infectious diseases, it is surprising that there are few books dedicated solely to this subject. Lisa Beltz has helped address this need with the first edition of Emerging Infections: A Guide to Diseases, Causative Agents, and Surveillance. The text’s stated objective is to fulfill the “… need for a college-level textbook in this field ...” and this goal was admirably attained.

The book comprises 30 chapters dealing primarily with selected microbial agents; 2 introductory chapters provide a useful basic summary of the scope of infectious diseases and emerging infections, as well as insights into host–agent interactions. Chapters on emerging infections in the immunocompromised host and on bioterrorism are also included. Each chapter is subdivided into 10 logical subunits that address the key aspects of each emerging infection. An initial major concepts section provides a chapter highlight and a bulleted summary, and key terms are included at the end of each chapter. Such repetition, which might seem redundant, is particularly effective at the undergraduate level.

Review questions and topics for further discussion are also provided. Although no references are included in the text, a series of resources is provided at the end of each chapter. The author borrows heavily from the excellent image library of the Centers for Disease Control and Prevention and this greatly enhances the book. Beltz has worked to make the material accessible, and the text is written largely in a colloquial and easy-to-understand fashion.

As would be expected in a first edition text covering such a broad topic, there are inevitable omissions and errata. It would have been useful to have expanded discussion, or perhaps added chapters, on zoonotic agents and their role in emerging infectious diseases, as well as additional information on the general principles of disease surveillance, and prevention and control approaches. There are minor misprints, such as the misspelling of cayatenensis, the use of syncytia (instead of syncytial), *Amoeba* (rather than *Entamoeba*) and humeral (instead of humoral). One notable omission is the absence of discussion of emerging helminthic diseases, such as angiostrongyliasis, cysticercosis, alveolar echinococcosis, and baylisascariasis. In addition, in an otherwise solid chapter on malaria, conspicuously absent was any discussion of the emergence of *Plasmodium knowlesi* in Southeast Asia. One could also argue for inclusion of different microbial agents than some of those presented, but I suspect that consensus on any such list would be unattainable.

Despite these drawbacks, Beltz has performed a fine job in producing a useful textbook on emerging infectious diseases that will work well in an upper-level undergraduate setting, as well as in courses for allied health professionals. Although this book might also be useful in graduate programs, supplementing it with journal articles and other sources will be necessary.

